# Supplementary material for: The First Pseudomonas Phage vB_PseuGesM_254 Active against Proteolytic Pseudomonas gessardii Strains
Source: Viruses. 2024 Sep 30;16(10):1561. doi: 10.3390/v16101561 (PMC11512268; doi:10.3390/v16101561)
Supplement: Supplementary file 1 [file viruses-16-01561-s001.zip › Table S4.pdf]

Table S4. Phage origin motifs prediction in the PseuGes_254 genome using Ori-Finder software

Motif BACKTTGTTCTTDATYTGTSWAGC

Start	P-value	Site		
41	1.37e-12	CGCAATGGTA	CACGTTTTTCTTGGTCTGTCAAGC	AAAATTTATT
10	5.09e-11	ATCTAGCTC	TCCTTTGTGCTTGATTTGAGCCGC	AATGGTACAC
277	1.13e-10	TGGATGTTAT	CACTATGTTCATTACTTGTCAAGC	TGAATTTTTA
336	1.17e-09	AAAAGGTGTT	GACGTGGTTTTTAATCCCTGTAGA	CTGAGCCACA

Motif AATAATTTATA

Start	P-value	Site		
211	1.60e-05	AATTTATATT	AATAATTTATA	TATATTTATA
198	1.60e-05	AATTTATATT	AATAATTTATA	TTAATAATTT
185	1.60e-05	AATTTATATT	AATAATTTATA	TTAATAATTT
172	1.60e-05	AATTTATATT	AATAATTTATA	TTAATAATTT
159	1.60e-05	AATTTATATT	AATAATTTATA	TTAATAATTT

Motif AGYSWCRTMAATCAAWSRCWGSKKAAC

Start	P-value	Site		
363	1.49e-16	CTGTAGACTG	AGCCACATCAATCAAACACAGGGGAAC	AAA
75	1.61e-14	AAAATTTATT	AGTGTCGTAAATCAATGGCTGCTTAAC	TATAGTATTA
