# Supplementary material for: The First Pseudomonas Phage vB_PseuGesM_254 Active against Proteolytic Pseudomonas gessardii Strains
Source: Viruses. 2024 Sep 30;16(10):1561. doi: 10.3390/v16101561 (PMC11512268; doi:10.3390/v16101561)
Supplement: Supplementary file 1 [file viruses-16-01561-s001.zip › Figure S1.pdf]

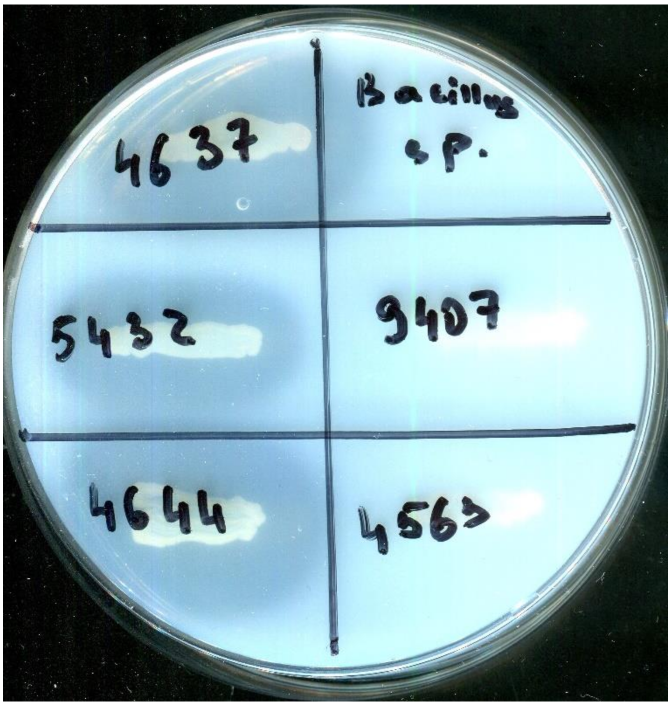

Figure S1. Bacterial strains sensitive to *Pseudomonas* phage PseuGes_254, namely *P. gessardii* CEMTC 4637, *P. gessardii* CEMTC 4644, and *P. gessardii* CEMTC 5432, exhibit proteolytic activity on milk agar (Skim milk 1.0%, Peptone 0.1%, NaCl 0.5%, Agar 2.0% and pH 8.0). Negative control of proteolysis: *Pseudomonas vancouverensis* CEMTC 4563, *Pseudomonas* sp. CEMTC 9407, and *Bacillus* sp isolate. Incubation at 25°C for 18 hours.
