# Supplementary material for: The First Pseudomonas Phage vB_PseuGesM_254 Active against Proteolytic Pseudomonas gessardii Strains
Source: Viruses. 2024 Sep 30;16(10):1561. doi: 10.3390/v16101561 (PMC11512268; doi:10.3390/v16101561)
Supplement: Supplementary file 1 [file viruses-16-01561-s001.zip › Table S1.pdf]

Table S1. List of phage genomes specific to bacterial strains from *P. fluorescens* group. Data extracted from the NCBI GenBank database

No	Phage host	Phage	Taxonomy	Genome ID, genome size
1	*P. chlororaphis*	Pseudomonas phage 201phi2	Caudoviricetes	NC_010821 316674 bp
2	*P. fluorescens*	Pseudomonas phage BIM BV-45	Caudoviricetes; *Autographiviridae; Bifseptvirus*	MT094430, 40565 bp
3	*P. fluorescens* group	Pseudomonas phage KNP	Caudoviricetes; *Autographiviridae; Studiervirinae; Ghunavirus; Ghunavirus KNP*	NC_047827, 40491 bp
4	*P. fluorescens*	Pseudomonas phage Pf1 ERZ-2017	Caudoviricetes; *Autographiviridae; Studiervirinae; Ghunavirus*	NC_047874, 39195 bp
5	*P. fluorescens group*	Pseudomonas phage WRT	Caudoviricetes; *Autographiviridae; Studiervirinae; Ghunavirus*	NC_047826, 40214 bp
6	*P. fluorescens* SBW25	Pseudomonas phage phi-2	Caudoviricetes; *Autographiviridae; Krylovirinae; Tunggulvirus; Tunggulvirus f2*	NC_013638 43144 bp
7	*P. fluorescens* strain 22	Pseudomonas phage phiGM22-3	Caudoviricetes; *Autographiviridae; Krylovirinae; Tunggulvirus*	MW627366, 42662 bp
8	*P. fluorescens* SW-3	Pseudomonas phage VSW-3	Caudoviricetes; *Autographiviridae; Napahaivirus; Napahaivirus VSW3*	NC_041885, 40556 bp
9	*P. fluorescens*	Pseudomonas phage Pf-10	Caudoviricetes; *Autographiviridae; Studiervirinae; Pifdecavirus; Pifdecavirus Pf10*	NC_027292, 39167 bp
10	*P. fluorescens*	Pseudomonas phage PFP1	Caudoviricetes; *Autographiviridae; Studiervirinae; Pifdecavirus*	NC_047997, 40914 bp
11	*P. fluorescens*	Pseudomonas phage Phi-S1	Caudoviricetes; Autographiviridae; Studiervirinae; Pifdecavirus	NC_021062 40192 bp
12	*P. fluorescens*	Pseudomonas phage BIM BV-46	Caudoviricetes; *Autographiviridae; Studiervirinae; Pifdecavirus*	MT094431, 38860 bp
13	*P. fluorescens*	Pseudomonas phage PCS4	Caudoviricetes; *Autographiviridae; Studiervirinae; Pifdecavirus*	OK094519, 39191 bp
14	*P. fluorescens*	Pseudomonas phage 22PfluR64PP	Caudoviricetes; *Autographiviridae; Studiervirinae; Pifdecavirus*	NC_047965, 40822 bp
15	*P. fluorescens*	Pseudomonas phage 67PfluR64PP	Caudoviricetes; *Autographiviridae; Studiervirinae; Pifdecavirus*	MH179478, 40748 bp
16	*P. fluorescens*	Pseudomonas phage 71PfluR64PP	Caudoviricetes; *Autographiviridae; Studiervirinae; Pifdecavirus*	MH179475, 40582 bp
17	*P. fluorescens*	Pseudomonas phage phiIBB-PF7A	Caudoviricetes; *Autographiviridae; Studiervirinae; Pifdecavirus*	NC_015264, 40973 bp
18	*P. fluorescens*	Pseudomonas phage UNO-SLW1	Caudoviricetes; *Autographiviridae; Studiervirinae; Pifdecavirus*	NC_047873 39215 bp
19	*P. fluorescens*	Pseudomonas phage UNO-SLW2	Caudoviricetes; *Autographiviridae; Studiervirinae; Pifdecavirus*	KX449361 39167 bp
20	*P. fluorescens*	Pseudomonas phage UNO-SLW3	Caudoviricetes; *Autographiviridae; Studiervirinae; Pifdecavirus*	KX449362 39092 bp
21	*P. fluorescens*	Pseudomonas phage UNO-SLW4	Caudoviricetes; *Autographiviridae; Studiervirinae; Pifdecavirus*	KX449363 39136 bp
22	*P. fluorescens*	Pseudomonas phage 98PfluR60PP	Caudoviricetes; *Schitoviridae; Littlefixvirus*	NC_070866, 74361 bp
23	*P. fluorescens* CS1	Pseudomonas phage phCDa	Caudoviricetes; *Schitoviridae; Shizishanvirus; Shizishanvirus phCDa*	NC_048003, 72821 bp

24	*P. fluorescens* SBW25	Pseudomonas phage Noxifer	Caudoviricetes; *Noxifervirus; Noxifervirus noxifer*	NC_041994, 278136 bp
25	*P. fluorescens* Pf1.1	Pseudomonas phage OBP	Caudoviricetes; *Petsuvirus*	NC_016571, 284757 bp
26	*P. fluorescens* ATCC 13525	Pseudomonas phage PMBT14	Caudoviricetes; *Knuthellervirus; Knuthellervirus PMBT14*	NC_048687, 47820 bp
27	*P. fluorescens*	Pseudomonas phage SCYZ1	Caudoviricetes; *Krylovvirus*	MH518298, 47475 bp
28	*P. fluorescens* SA1	Pseudomonas phage PPSC2	Caudoviricetes; *Otagovirus*	NC_073678 97330 bp
29	*P. fluorescens*	Pseudomonas phage UFV-P2	Caudoviricetes; *Vicosavirus; Vicosavirus UFVP2*	NC_018850 45517 bp
30	*P. fluorescens*	Pseudomonas phage Phabio	Caudoviricetes	NC_062582, 309157 bp
31	*P. fluorescens* SBW25	Pseudomonas phage Skulduggery	Caudoviricetes	NC_070827, 62978 bp
32	*P. fluorescens* UFV 041	Pseudomonas phage UFJF_PfDIW6	Caudoviricetes	NC_070881 42322 bp
33	*P. fluorescens*	Pseudomonas phage VW-6B	Caudoviricetes	MF975721 35306 bp
34	*P. fluorescens*	Pseudomonas phage VW-6S	Caudoviricetes	MF975720 37917 bp
35	*P. koreensis*	Pseudomonas phage AH02	Caudoviricetes	MZ501271, 39095 bp
36	*P. protegens* CEMTC 4060	Pseudomonas phage PseuP_222	Caudoviricetes	OP626800 56717 bp
37	*P. protegens* CEMTC 4060	Pseudomonas phage PseuP_224	Caudoviricetes	OP795451 43182 bp
