# Supplementary material for: The First Pseudomonas Phage vB_PseuGesM_254 Active against Proteolytic Pseudomonas gessardii Strains
Source: Viruses. 2024 Sep 30;16(10):1561. doi: 10.3390/v16101561 (PMC11512268; doi:10.3390/v16101561)
Supplement: Supplementary file 1 [file viruses-16-01561-s001.zip › Table S2.pdf]

Table S2. Bacterial isolates used to determine the host range of the bacteriophage PseuGes_254

No	CEMTC ID	**Species according 16S rRNA gene**	Isolation source	GenBank Accession, 16S rRNA
1	3370	*Pseudomonas gessardii*	water and sediments, Lake Geizernoe, Altai Republic	PP439619
2	3899[1]	*Pseudomonas proteolytica*	coastal soil, The Chukchi Peninsula, Arctica	PP874608
3	**4637**[1]	*Pseudomonas gessardii*	water and sediments, Chemal river, Altai Republic	ON838145
4	**4644**[1]	*Pseudomonas gessardii*	water and sediments, Chemal river, Altai Republic	OQ834588
	4787	*Pseudomonas synxantha*	*Leptinotarsa decemlineata*, Novosibirsk, Russia	PP874613
6	4826	*Pseudomonas synxantha*	*Leptinotarsa decemlineata*, Novosibirsk, Russia	PP874614
7	4877	*Pseudomonas yamanorum*	*Leptinotarsa decemlineata*, Novosibirsk, Russia	PP874619
8	5408[1]	*Pseudomonas brenneri*	water and sediments, Chuya river, Altai Republic	PP874583
9	**5432**[1]	*Pseudomonas gessardii*	water and sediments, Inegen river, Altai Republic	PP348772
10	6787	*Pseudomonas gessardii subgroup*	garden soil, Novosibirsk region	-
11	7127[1]	*Pseudomonas gessardii*	feces of *Bos grunniens*, Kyrgyzstan republic	PP439620
12	9055[1]	*Pseudomonas gessardii*	water and sediments, Dzhazator river, Altai Republic	PP439621
13	9325[1]	*Pseudomonas gessardii*	water and sediments of the no name lake,Ulagan plateau, Altai Republic	PP439622
14	9334[1]	*Pseudomonas gessardii*	water and sediments, Gull Lake, Novosibirsk region	PP439623
15	9351[1]	*Pseudomonas synxantha*	water and sediments, Kamnevo Lake, Novosibirsk region	PP874615
16	9481	*Pseudomonas brenneri*	water and sediments, water reservoir, Novosibirsk, Russia	PP874584
17	9531	*Pseudomonas brenneri*	water of a mountain glacier, Altai Republic	PP874585
18	9534	*Pseudomonas meridiana*	water of a mountain glacier, Altai Republic	PP874601
19	9742	*Pseudomonas gessardii*	water and sediments, Lake Aflatun, Kyrgyzstan republic	PP874590

Bacterial strains sensitive to phage PseuGes_254 are marked with green boxes and bold type; [1] – strains used for taxonomy clarification
