# Supplementary material for: The First Pseudomonas Phage vB_PseuGesM_254 Active against Proteolytic Pseudomonas gessardii Strains
Source: Viruses. 2024 Sep 30;16(10):1561. doi: 10.3390/v16101561 (PMC11512268; doi:10.3390/v16101561)
Supplement: Supplementary file 1 [file viruses-16-01561-s001.zip › Table S3.pdf]

Table S3. Primers for PCR and sequencing

Target genes	Primer sequences (5′ → 3′)	The size of the PCR product, bp	Annealing temperature	Reference
16S rRNA	8F: AGRGTTTGATCCTGGCTCA<br>1350R: GACGGGCGGTGTGTACAAG	1308	55°C	[37]
rpoD	rpoD-F: ACTTCCCTGGCACGGTTGACCA<br>rpoD-R: TCGACATGCGACGGTTGATGTC	693	60°C	[10]
rpoB	rpoB-F: CAGTTCATGGACCAGAACAACCCGCT<br>rpoB-R: CCCATCAACGCACGGTTGGCGTC	483	58°C	[10]
gyrB	gyrB-F: TTCAGCTGGGACATCCTGGCCAA<br>gyrB-R: TCGATCATCTTGCCGACRACCA	564	65°C	[10]
