# Supplementary figures and images for: The First Pseudomonas Phage vB_PseuGesM_254 Active against Proteolytic Pseudomonas gessardii Strains
Source: Viruses. 2024 Sep 30;16(10):1561. doi: 10.3390/v16101561 (PMC11512268; doi:10.3390/v16101561)

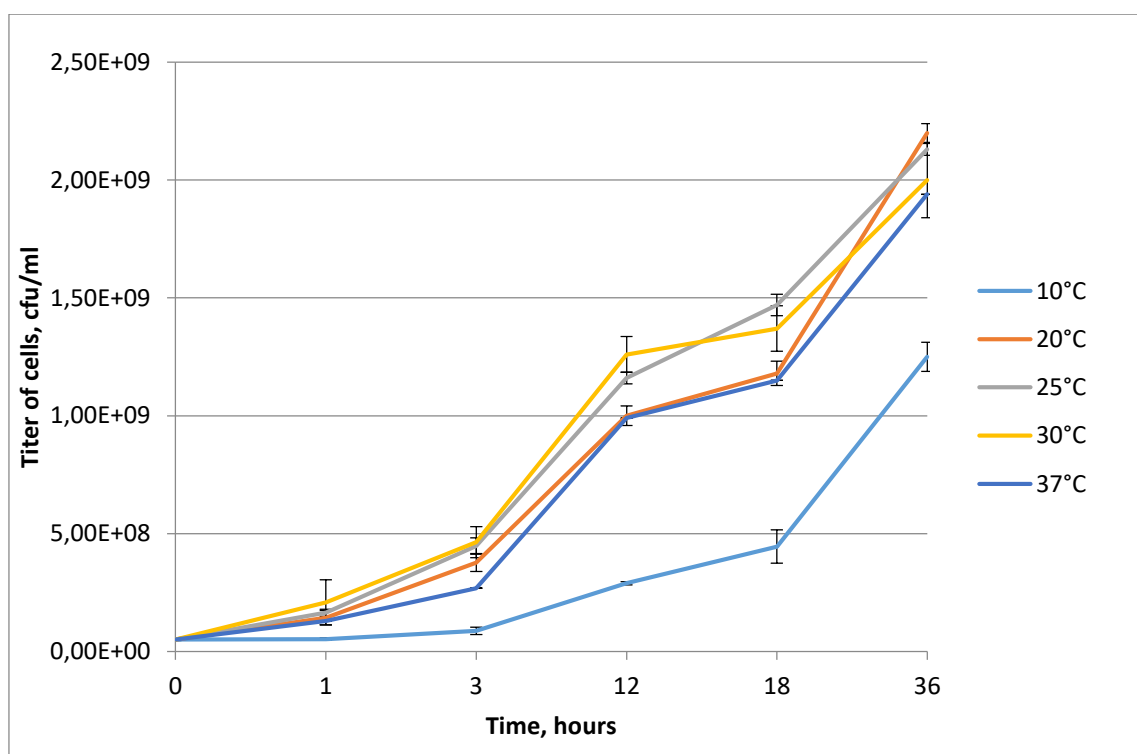

Supplement: Supplementary file 1 [file viruses-16-01561-s001.zip › Figure S2.pdf]

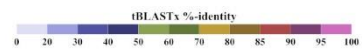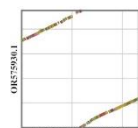

CALTWU010000032.1  
CALTWU010000032.1  
46,611 nt

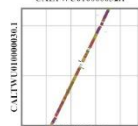

OR575930.1  
OR575930.1  
95,072 nt

CALTWU010000030.1  
CALTWU010000030.1  
46,990 nt

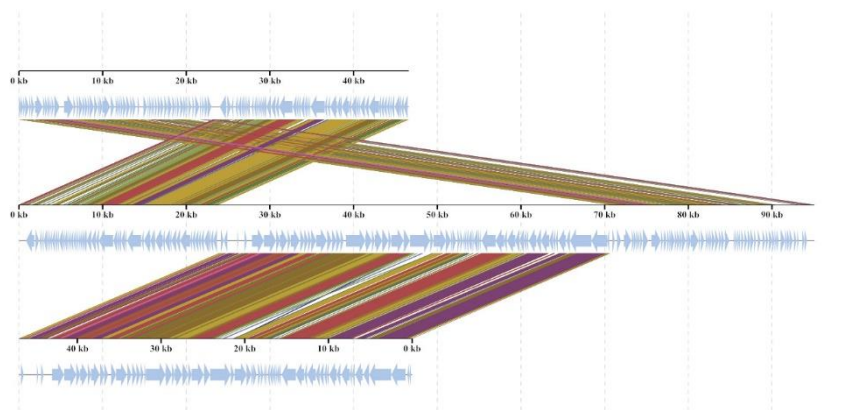

Supplement: Supplementary file 1 [file viruses-16-01561-s001.zip › Figure S4.pdf]

A

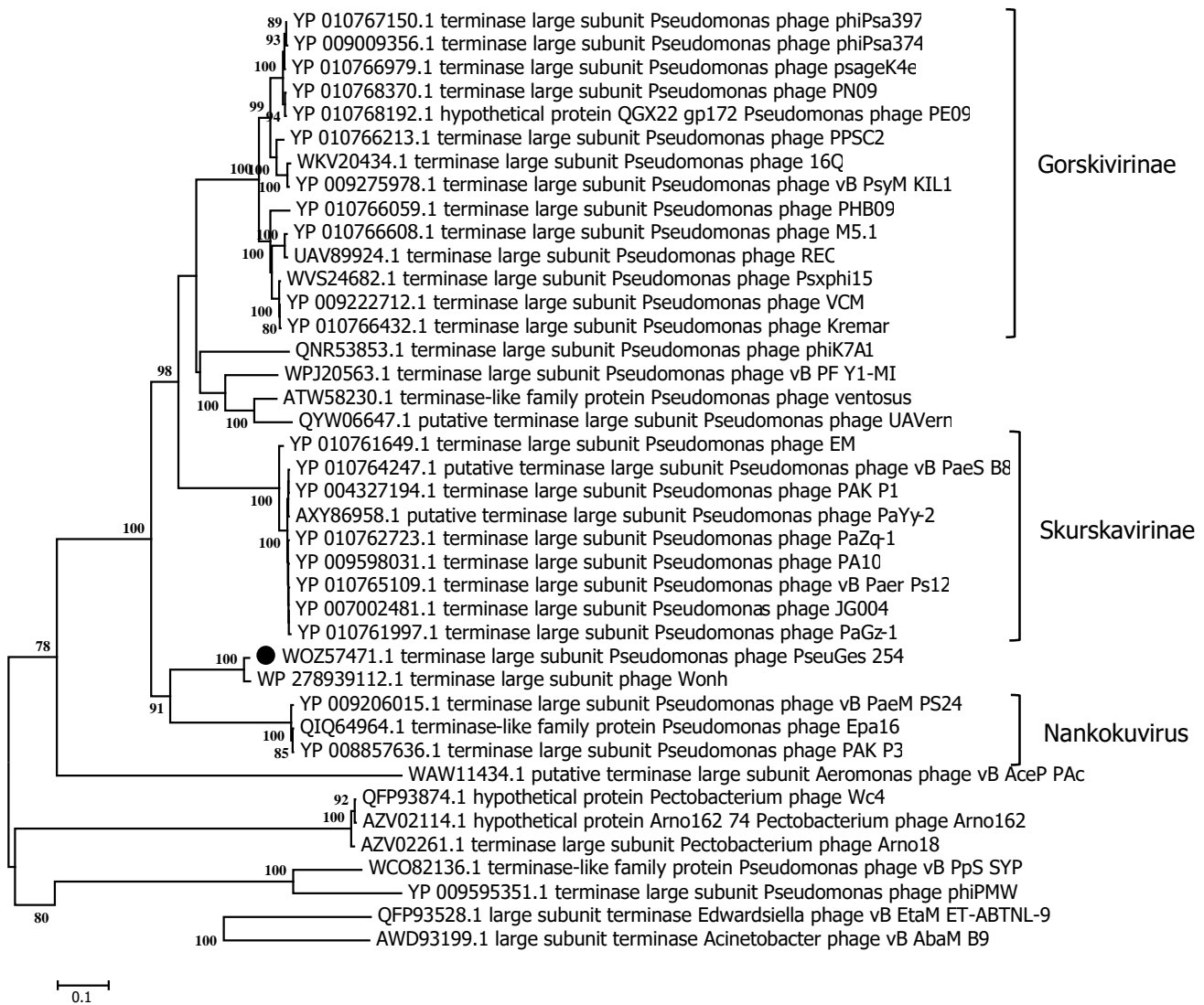

B

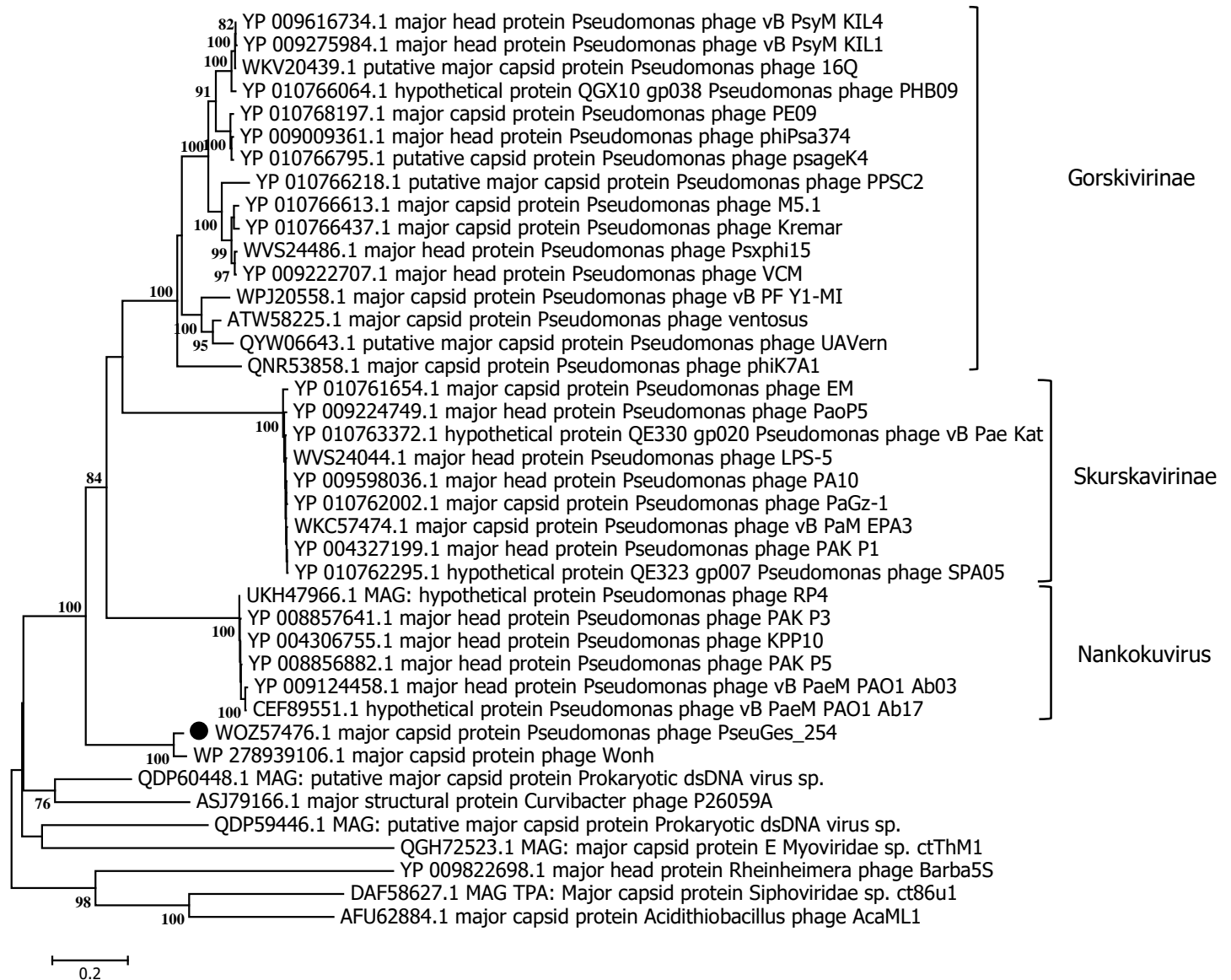

Supplement: Supplementary file 1 [file viruses-16-01561-s001.zip › Figure S5.pdf]
